# Amazonian scorpions and scorpionism: integrating toxinological, clinical, and phylogenetic data to combat a human health crisis in the world’s most diverse rainforest

**DOI:** 10.1590/1678-9199-JVATITD-2021-0028

**Published:** 2021-11-29

**Authors:** Adolfo Borges, Matthew R. Graham, Denise M. Cândido, Pedro P. O. Pardal

**Affiliations:** 1 Center for the Development of Scientific Research (CEDIC), Asunción, Paraguay.; 2 Laboratory of Molecular Biology of Toxins and Receptors, Institute of Experimental Medicine, School of Medicine, Central University of Venezuela, Caracas, Venezuela.; 3 Department of Biology, Eastern Connecticut State University, Willimantic, CT, United States.; 4 Laboratory of Arthropods, Butantan Institute, São Paulo, SP, Brazil.; 5 Laboratory of Medical Entomology and Venomous Animals, Center of Tropical Medicine, Federal University of Pará (UFPA), Belém, PA, Brazil.

**Keywords:** Amazonia, Scorpionism, Scorpion antivenom, Tityus

## Abstract

Venom from Amazonian scorpions of the genus *Tityus* contains components capable of eliciting a distinct clinical, mostly neurological, syndrome. This contrasts with the mainly autonomic manifestations produced after envenomation by congeneric southern and northern South American species. Herein, we summarize Pan-Amazonian scorpionism by synthesizing available toxinological, clinical, and molecular data gathered from all affected areas in Amazonia, including Brazil, Ecuador, Colombia, Peru, Venezuela, and French Guiana. We searched multiple databases, as well as our own records, for reports of scorpion envenomations in Amazonia by confirmed *Tityus* spp., and compared the clinical manifestations. To help uncover clinical and venom relationships among problematic species, we explored phylogenetic relationships with a rate-calibrated analysis of mitochondrial COI data from available species. The possible existence of diversity gradients for venom toxic and immunogenic components despite the predicted strong phylogenetic association among species is underscored by discussed clinical and toxinological findings. A multicentric effort, involving all nations affected by this neglected disease, is urgently needed to offer alternatives for treating and understanding this pathology, including the preparation of neutralizing antibodies with a broad range of efficacy.

## Background

Scorpionism, or the medical consequence of scorpion stings in humans, is a neglected health problem in tropical and subtropical areas associated with poverty and the lack of access to effective antivenoms. Rapid tissue distribution of scorpion toxins targeting specific ion channels associated with excitable and immunological cells usually results in high mortality rates in children under 10 years of age. As such, severe stings require prompt treatment with specific antivenoms and intensive cardiorespiratory support [1]. In the Americas, Mexico has the highest scorpion envenomation incidence [2], followed by the Amazon region where the rate has been estimated to range between 30 and 200 cases per 100,000 inhabitants [3]. These numbers are most probably an underestimation as large sections of Amazonia remain epidemiologically underreported, with the number of cases to be much higher in remote riverine and indigenous communities [3-5].

*Tityus* represents a diverse group of buthid scorpions primarily distributed throughout South America, Central America, and the Caribbean. Species of the genus are responsible for the majority of severe envenomation cases throughout South America, especially the Amazon region, where it reaches its highest species diversity [5, 6]. Analyses of *Tityus* envenomations throughout Amazonia, mainly Brazil, have revealed neurological manifestations that sharply contrast with the mainly peripheral manifestations elicited by congeneric species from northern and southeastern South America [6]. Thus, Amazonian *Tityus* contain venoms with unique physiopathological mechanisms.

Significant efforts have been made to understand and treat scorpionism in Amazonia, particularly along the Brazilian Amazon River Basin [4, 7, 8]. In this region, a pattern of increased scorpion sting incidence is notable from 2000 to 2017, especially in the states of Pará, Tocantins, Maranhão and Mato Grosso. Lethality from stings in these areas is significantly higher compared to other regions of Brazil, probably due to a lack of experienced health personnel, appropriate antivenom-based therapies, and an overall lower quality of care in rural towns [5]. A research consortium “Snakebite and Scorpionism Network in the Amazon” has emerged as a joint effort from scientists at the Butantan Institute and the Tropical Medicine Foundation, in Manaus, to understand and combat the problem in Brazil [4]. However, a similar pathology occurs in other regions of the Amazon Basin as well. Specifically, severe cases and fatalities have been reported from French Guiana, Venezuela, Guyana, Colombia, Ecuador, and Peru [9-16].

**Figure 1.**
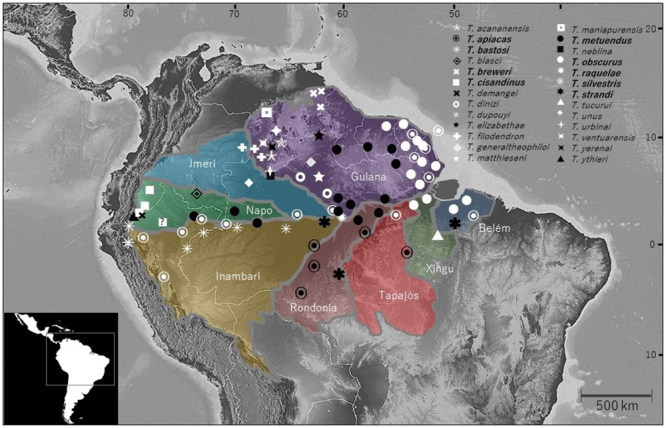
Distribution map of representative Amazonian *Tityus* species, superimposed on the areas of endemism defined for Amazonia [17]. Distribution areas for Brazilian, Colombian, Ecuadorean, Peruvian, and French Guiana taxa [20, 23, 29, 30, 31, 32, 33] and distribution areas for Venezuelan taxa [27, 28]. *T. cisandinus* distribution in Peru has been posited but not demonstrated [34]. Species names in boldface correspond to medically important species (see Table 1).

Amazonia is a mosaic of eight areas of endemism (Figure 1), which share ecologically similar characteristics, but delineated by the distributions of co-distributed taxa, including scorpions [17-20]. Our understanding of scorpionism in this region would undeniably benefit from a comparative analysis of data on the distributions of medically significant species (*Tityus* spp*.*), envenomation physiopathology, and toxinology. Additionally, relationships among Amazonian *Tityus* and their venoms could be improved by molecular phylogenetic analyses.

About 65% of Amazonia lies within Brazil, but only four areas of endemism are almost entirely (Rondônia) or entirely (Tapajós, Xingu, and Belém) Brazilian. Less than 50% of the Napo and Imeri areas are in Brazil, and the scorpion envenomation problem is increasing in sections of these areas in Colombia, Ecuador and Peru [14, 16] (see also https://web.ins.gob.pe/index.php/es/prensa/noticia/instituto-nacional-de-salud-traslado-suero-antiescorpionico-para-nino-de-comunidad).

Recently, a phylogeny generated with mitochondrial markers revealed that *T. cisandinus* from Amazonian Ecuador (Napo area) is closely related to medically significant *T. obscurus* populations from the Brazilian northeast. Thus, species capable of severe envenomations, the putative “*Tityus obscurus*” species complex, are distributed across the Amazon basin [14], a result corroborated by recent morphological data [20]. A joint effort by scorpion biologists and toxinological/medical teams should help elucidate the actual extent of this and other species complexes in Amazonia. Such an understanding will lay the framework for studies of shared and/or expression of unique venom components, and will aid in the design of better therapeutic tools against scorpionism.

This review integrates clinical and toxinological data on scorpionism and scorpions from the regions of endemism that comprise Amazonia. The territories of Guyana, Surinam, French Guiana, and southeast Venezuela do not belong to the Amazon River Basin, but they share problematic scorpion species with other areas of the Basin. As such, they will be considered as part of Amazonia in this review, providing a Pan-Amazonian perspective on scorpionism. Additionally, relationships among Amazonian *Tityus* spp. of medical importance are explored within a molecular phylogenetic context. We hope this review encourages further investigation across all countries involved in the search of collective alternatives to study and combat scorpionism across Amazonia.

##  Towards a working phylogeny of Amazonian Tityus 

About 2% of the world´s arachnids live in Amazonia, and almost 25% of the arachnid families presently known are represented in this region. Of these, about 200 species are scorpions, comprising about 13% of the world´s scorpion diversity [21]. Despite this diversity, only four of the Neotropical scorpion families are represented in the Amazon: Buthidae, Chactidae, Ischnuridae, and Troglotayosicidae [22]. Of these, Buthidae is by far the most diverse, with most species belonging to the genus *Tityus*, which cause most severe human envenomations in the region [23]. Clinical data are lacking for the other Amazonian scorpions, suggesting *Tityus* spp. may be the only regional scorpions for which stings result in more than just local symptomatology. The large and abundant chactid *Brotheas amazonicus*, for example, is known to invade disturbed areas but is barely toxic to mice [24].

**Figure 2.**
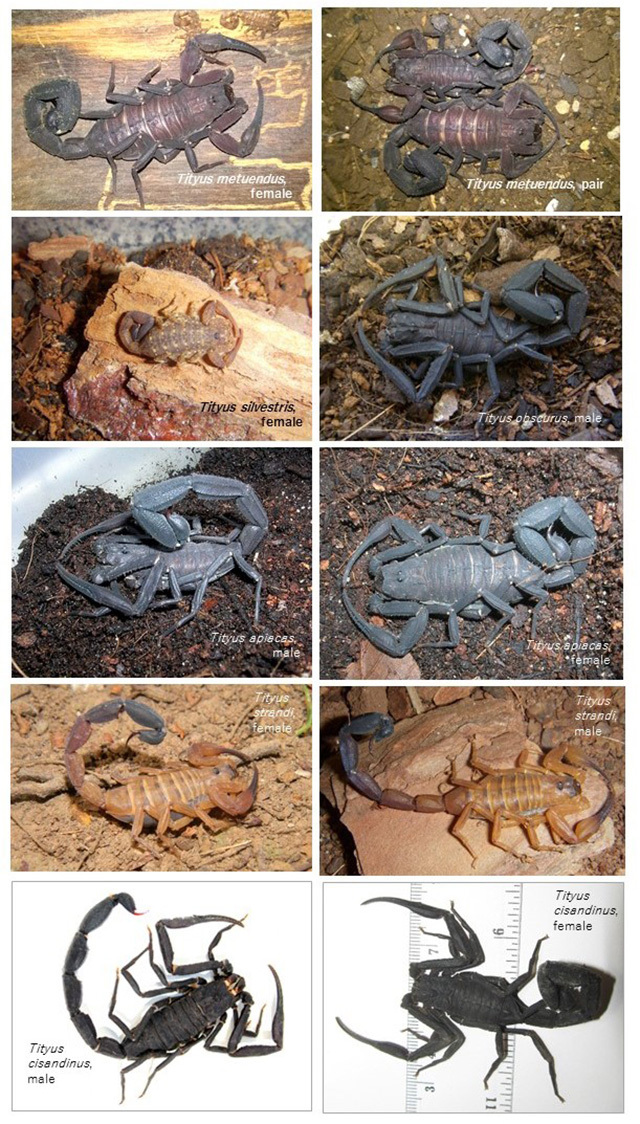
Representative *Tityus* species from Brazilian and Ecuadorean Amazonia. Photographs of Brazilian specimens by Denise Cândido, and those of *T. cisandinus* by Adolfo Borges.

To date, a total of 49 *Tityus* spp. have been reported from the eight abovementioned areas of endemism (Table 1). These species represent 22% of the known *Tityus* diversity (n = 224) [25], and inhabit eight Amazonian countries: Colombia, Ecuador, Peru, Venezuela, Guyana, French Guiana, Suriname, and Brazil. A map of the Amazon region depicting approximate distributions for some of these species, superimposed on the reported areas of endemism [17], is provided in Figure 1. The habitus of representative *Tityus* species are presented in Figure 2, and medically important taxa from Brazilian and Ecuadorean Amazonia are listed in Table 1 (in bold).

**Table 1.** Updated list (as of January 2021) of Amazonian species (n = 49) in the genus *Tityus* and their distribution in Amazonian areas of endemism [17]. Species from Brazilian Amazonia are listed based on Monteiro et al. [5], with updates from Lourenço et al. [20]. Species from French Guiana are listed based on Ythier [26], species from Venezuela are based on Ochoa and Rojas-Runjaic [27] and González-Sponga [28], and records from Colombia according to Lourenço [29]. Species in bold correspond to taxa implicated in reported envenomation cases [5, 15, 14].

Species	Distribution	Amazonian subregion
*Tityus acananensis* González-Sponga, 2009	Acanaña, Alto Orinoco municipality, Amazonas state, Venezuela	Guiana
*Tityus adisi* Lourenço & Pezier, 2002	Tarumã Mirim, Manaus region, Amazonas state, Brazil	Guiana
*Tityus anduzei* González-Sponga, 1997	Miyayobaweteri, Parima range, Atabapo department, Amazonas state, Venezuela	Guiana
*Tityus anori* Lourenço, Rossi & Wilmé, 2019	Anori, Amazonas state, Brazil	Guiana
***Tityus apiacas* Lourenço, 2002**	Northern Mato Grosso, Rondônia, southern Amazonas and Pará states, Brazil	Inambari, Tapajós, Rondônia
*Tityus bastosi* Lourenço, 1984	Central and eastern Amazonia, spanning Brazil, Perú, Colombia, and Ecuador	Guiana
***Tityus breweri* González-Sponga, 1997**	Northeastern Bolívar state, Venezuela; possibly Guyana	Inambari
*Tityus blanci* Lourenço, 1994	Amazonas and Meta departments, Colombia	Napo
*Tityus canopensis* Lourenço, 2002	Tarumã Mirim, Manaus region, Amazonas state, Brazil	Guiana
*Tityus cesarbarrioi* González-Sponga, 2001	Santa Rosa creek, Cuyuní river basin, Bolívar state, Venezuela	Guiana
***Tityus cisandinus* Lourenço & Ythier, 2017**	Morona Santiago and Pastaza provinces, Ecuador; possibly Loreto department, Perú	Napo
*Tityus clathratus* C.L. Koch, 1844	Roraima state, Brazil; Guyana	Guiana
*Tityus culebrensis* González-Sponga, 1994	Culebra, Atabapo department, Amazonas state, Venezuela	Guiana
*Tityus demangei* Lourenço, 1981	Los Tayos cave, Morona Santiago province, Ecuador	Napo
*Tityus dinizi* Lourenço, 1997	Amazonas state, Brazil	Guiana
*Tityus dupouyi* González-Sponga, 1987	Simarawochi, Atabapo department, Amazonas state, Venezuela	Guiana
*Tityus elizabethae* Lourenço & Ramos, 2004	Marco Brasil Venezuela Nº 8, Pacaraima, Roraima state, Brazil	Guiana
*Tityus filodendron* González-Sponga, 1981	Río Negro department, Amazonas state, Venezuela; Guainía department, Colombia	Guiana, Imeri
*Tityus gasci* Lourenço, 1981	French Guiana; Amazonas and Roraima states, Brazil; Perú	Guiana
*Tityus generaltheophiloi* Lourenço, 2017	Serra da Mocidade, Roraima state, Brazil	Guiana
*Tityus grahami* Lourenço, 2012	Barcelos, Upper Rio Negro, Amazonas state, Brazil	Guiana
*Tityus jussarae* Lourenço, 1988	Alto Lagarto cave, Napo province, Ecuador	Napo
*Tityus lokiae* Lourenço, 2005	Amazonas state, Brazil	Guiana
*Tityus kukututee* Ythier, Chevalier & Gangadin, 2020	Pierre Kondre, near Carolina, Para District, Suriname	Guiana
*Tityus mana* Lourenço, 2012	Central and northeastern French Guiana	Guiana
*Tityus manakai* González-Sponga, 2004	Manaka, Atabapo municipality, Amazonas state, Venezuela	Guiana
*Tityus maniapurensis* González-Sponga, 2009	Los Colorados, Cedeño municipality, Bolívar state, Venezuela	Guiana
*Tityus matthieseni* Pinto-da-Rocha & Lourenço, 2000	Roraima state, Brazil	Guiana
*Tityus marajoensis* Lourenço & da Silva, 2007	Pará state, Brazil	Belém
***Tityus metuendus* Pocock, 1897**	Amapá, Pará, Roraima, and Amazonas states, Brazil; Loreto department, Perú; Sipaliwini District, Suriname; Rupununi River next to Lethem, Guyana	Guiana, Rondônia, Inambari, Napo
*Tityus neblina* Lourenço, 2008	Neblina Peak, border between Brazil and Venezuela	Guiana
*Tityus nelsoni* Lourenço, 2005	São Gabriel da Cachoeira, Río Negro region, Amazonas state, Brazil	Imeri
***Tityus obscurus* (Gervais, 1843)**	Amapá and Pará states, Brazil; widespread in French Guiana; Suriname	Guiana, Belém, Tapajós. Xingú
***Tityus raquelae* Lourenço, 1988**	Amazonas state, Brazil	Guiana
*Tityus riocaurensis* González-Sponga, 1996	Tabaro River, Rio Caura Forest Reserve, Cedeño municipality, Bolívar state, Venezuela	Guiana
*Tityus rionegrensis* Lourenço, 2006	Amazonas state, Brazil	Guiana
*Tityus sarisarinamensis* González-Sponga, 2002	Jaua-Sarisariñama National Park, Sucre municipality, Bolívar state, Venezuela	Guiana
*Tityus romeroi* González-Sponga, 2008	El Palmar, Imataca range, Bolívar state, Venezuela	Guiana
*Tityus shiriana* González-Sponga, 1991	Neblina Peak, Río Negro department, Amazonas state, Venezuela	Guiana
***Tityus silvestris* Pocock, 1897**	Widespread along the Amazon basin spanning Brazil, Ecuador, and Perú	Guiana, Belém, Tapajós. Inambari, Napo
***Tityus strandi* Werner, 1939**	Pará and Amazonas states, Brazil, along the Amazon and Solimões river basins	Belém, Inambari
*Tityus sylviae* Lourenço, 2005	PNJ Seringalzinho, Río Negro region, Amazonas state, Brazil	Imeri
*Tityus tucurui* Lourenço, 1988	Cenral and eastern Pará state, Brazil	Imeri
*Tityus unus* Pinto-da-Rocha & Lourenço, 1984	Tapurucuara, Amazonas state, Brazil	Xingú
*Tityus urbinai* (Scorza, 1954)	Mawari-Anejidi, Atabapo department, Amazonas state, Venezuela	Guiana
*Tityus venamensis* González-Sponga, 1981	Venamo Hill, Roscio district, Bolívar state, Venezuela	Guiana
*Tityus ventuarensis* González-Sponga, 2009	Juanaña, border between Atures and Atabapo municipalities, Amazonas state, Venezuela	Guiana
*Tityus yerenai* González-Sponga, 2009	Caño Iguana, Manapiare municipality, Amazonas state, Venezuela	Guiana
*Tityus ythieri* Lourenço, 1988	South of Yaupi, Morona Santiago province, Ecuador	Napo

After reviewing the distributional data, *T. metuendus*, *T. obscurus*, *T. silvestri*s, *T. bastosi*, *T. apiacas*, and *T. strandi* are clearly the most widely distributed species in the Amazon Basin. These are also responsible for the majority of envenomation cases in Brazilian Amazonia [5]. However, most Amazonian *Tityus* species are only known from their type localities, and little information is currently available on their actual areas of distribution. Data presented in Figure 1 and Table 1 show that Guiana harbors the greatest number of species (n = 36), followed by Napo (n = 5), Imeri (n = 4), Inambari (n = 4), Belém (n = 3), Tapajós (n = 3), Rondônia (n = 2), and Xingú (n = 2). Large sections of Amazonia, mainly in Inambari and Imeri, remain poorly sampled. More thorough sampling of these areas may result in the discovery of new taxa. Therefore, we suspect that the number of medically important *Tityus* species across Amazonia, mainly belonging to the subgenus *Atreus*, is probably higher [23], and additional species, particularly in rainforest areas of Peru and northern Bolivia, are probably yet to be discovered.

The absence of phylogenetic analyses of *Tityus* species in Amazonia have hindered efforts to understand toxinological relationships among problematic species [30]. Only two phylogenetic analyses of *Tityus* spp. have been published; one encompassing Venezuelan species [31], and another comprising the southernmost South American species [32]. Assuming that there is some phylogenetic signal to venom toxicity in *Tityus* (i.e venoms are more similar among closely related species), as indicated by recent studies [33], then phylogenetic data can be used to make predictions regarding the toxicity of species that have yet to be toxinologically assessed.

**Figure 3.**
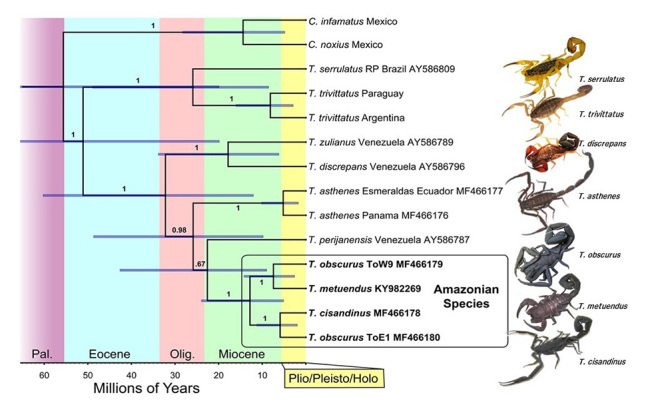
Bayesian chronogram of medically significant *Tityus* spp. in South America, generated in BEAST. Values at nodes indicate posterior probabilities; bars indicate highest posterior density (HPD) values around mean date estimates. GenBank accession numbers are provided in the tip labels. *Centruroides infamatus* and *C. noxius* (Buthidae) were included as an outgroup. Samples of *T. obscurus* ToW9 and ToE1 come from western and eastern Pará, Brazil, respectively.

Divergence time estimates, based on mitochondrial COI (cytochrome oxidase subunit I) data used in DNA barcoding [31], are provided in Figure 3 for 11 medically important *Tityus* species in South America; this includes samples of Amazonian *T. obscurus* (two populations, from eastern and western Pará, Brazil [14]), *T. metuendus* (from the Essequibo area in Guyana [34]), and *T. cisandinus* (from Morona Santiago, Ecuador [14]). Sequences for these species, as well as two outgroup samples (*Centruroides infamatus* and *C. noxius*) were retrieved from GenBank and aligned in Geneious v.7.1.7 (Biomatters Ltd., Auckland, New Zealand) using MUSCLE [35]. We estimated the best-fit substitution model with MEGA X [36] and conducted a Bayesian analysis in BEAST 1.8.0 [37]. We performed two independent MCMC runs for 40 million generations each and sampled every 4,000 generations, with a uncorrelated lognormal clock model, Yule tree prior, and mean rate (ucld.mean) adjusted according to clock calibrations used in previous analyses of *Tityus* scorpions [32].

The Bayesian analysis identified two major groups within *Tityus*: a clade comprising southern South American species (*T. serrulatus* and *T. trivittatus* populations from Paraguay and Argentina), and a second group incorporating species from Lower Central America (*T. asthenes*), northern South America (*T. discrepans, T. zulianus, T. perijanensis*), and Amazonian species *T. obscurus, T. metuendus*, and *T. cisandinus*. Interestingly, the three Amazonian species were rendered as monophyletic with strong support. The two main clades have been reported in previous studies [31, 32], but ours is the first to document the common ancestry of Amazonian *Tityus.*

Although node support was not strong, our phylogenetic analyses suggest that the Colombian/Venezuelan *T. perijanensis* is sister to the Amazonian species (Figure 3). This association is supported by data on *T. perijanensis* envenomation syndrome (e.g presence of neurological manifestations in envenomed humans such as tonic-clonic convulsions and neurogenic bladder) and toxinology (e.g sharing of toxin homologs with the Amazonian group) [33, 38]. Divergence time estimates indicate that the Amazonian populations probably shared a common ancestor between the early and late Miocene, with a middle Miocene mean estimate of approximately 13 Ma. Subsequent diversification among our samples is estimated to have occurred during the late Miocene and Pliocene. An examination of pairwise distance values, uncorrected and KTP corrected (Additional file 1), supports the distinction of the Amazonian samples, as nucleotide diversity is significantly less among them than among the non-Amazonian *Tityus* species (*t*-test p < 0.001).

Considering the closer relationships among the Amazonian *Tityus* species, despite their wide distribution (see *T. obscurus* and *T. metuendus* in Figure 1), we predict that other species that are morphologically similar may be members of the clade as well; particularly *T. apiacas*, *T. dinizi*, and *T. tucurui* [20]. The inclusion of *T. cisandinus* from Ecuador as a member of the Amazonian clade was recently demonstrated by our team [14].

Results from current analysis together with venom studies on *Tityus* spp. [33], indicate that species from Lower Central America, Colombia, and the Amazonian regions of Brazil, Ecuador, Peru, and French Guiana, might share enough molecular and toxinological similarities to justify preparation of a scorpion antivenom that should neutralize common venom toxic components. Venom gland transcriptomic data from medically important Amazonian scorpions could confirm this prediction, as the availability of toxin primary structures, deduced from their corresponding cDNAs, should enable toxin comparisons between species and the mapping of antigenic epitopes, as in the case of *Tityus serrulatus* [39]. Of the Amazonian species of medical importance, the phylogenetic position of *T. silvestris* would be of particular interest. The species is currently classified in subgenus *Archaeotityus* [40], which comprises small (18-40 mm), variegated scorpions, known to be of little medical significance outside of Amazonia [41]. Within the Amazon River Basin, however, *T. silvestris* has caused serious envenomations in Brazil, with presentation of neurological manifestations (see section 3). Molecular phylogenetic analyses of other scorpions in subgenus *Archeotityus* have grouped them in a clade that is sister to southern South American *Tityus* [32, 31]. Interestingly, *T. clathratus*, the type species of this subgenus, produces neurotoxins that are structurally related to β-toxins targeting sodium channels from congeneric southern South America species of medical significance [41].

##  Medical importance of Amazonian Tityus spp.: clinical manifestations and implications associated with envenomation by accurately identified species 

Tackling the scorpionism problem in Amazonia, particularly in the case of toxic *Tityus* fauna, relies on the identification of specimens associated with envenomations and the correct geographical delimitation of high-risk areas. Secondly, this approach should help uncover shared/differential clinical trends among toxic species, which is essential to the design of new therapeutic tools like the manufacture of a scorpion antivenom effective throughout Amazonia. Ideally, appropriate therapeutic measures should be based on the accurate identification of scorpions responsible for stings, as the envenomation syndrome is dependent on the species. This has been demonstrated with *Tityus* spp. from Amazonian (predominant neurological manifestations) versus southeastern South American (predominantly peripheral manifestations) species, and also in the case of envenomation by the Venezuelan *T. discrepans* (mainly gastrointestinal alterations) and *T. zulianus* (mainly cardiorespiratory manifestations) [6].

Historically, insufficient efforts have been made to accurately identify *Tityus* spp. implicated in medically significant envenomations [23]. Fortunately, physicians have recently started working with scorpion biologists to identify taxa associated with such incidents [8, 14, 30, 42, 43, 44, 45, 46]. This is an important approach, as we now understand that the composition and physiological activity of *Tityus* venoms vary substantially across the geographic distribution of the genus [33, 47, 48]. This result is not surprising, however, given the evolutionary complexity of the genus, the most diverse of all scorpion genera [25].

**Figure 4.**
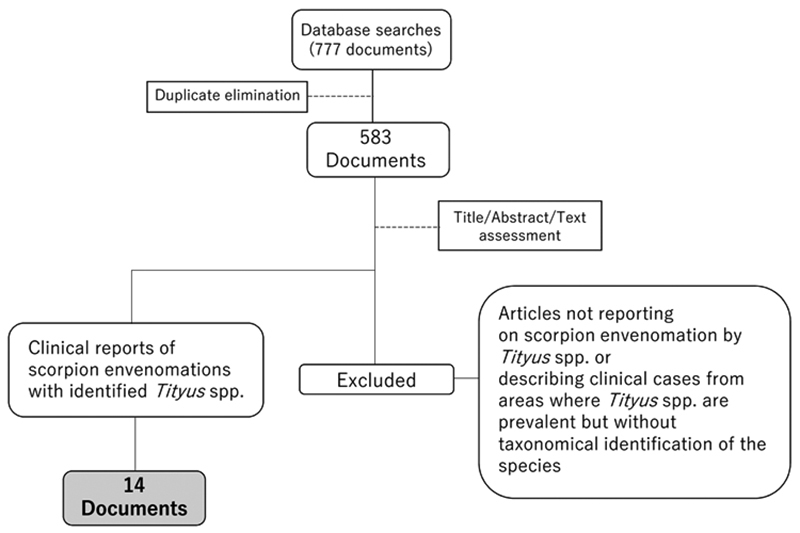
Flow diagram of the review for selecting reports on scorpion envenomation cases from Amazonia caused by taxonomically confirmed *Tityus* spp. for further analysis of clinical manifestations.

Given the complexity of Amazonian *Tityus*, we decided to assess published reports on envenoming syndromes in the region that accurately identified the scorpion as a *Tityus* spp. following the methodology suggested by PRISMA guidelines [49]. We searched Medline, Scopus, ISI Web of Knowledge, LILACS, and SciELO; the query argument was “Amazonian AND *Tityus* AND Scorpion AND (Sting OR Envenomation)”. Searches retrieved records from 1950 to 2020, including additional documents identified by searching bibliographies of the retrieved studies and in the authors’ records. This search strategy aimed at recovering documents clinically describing envenomation cases in Amazonia where the responsible scorpion species was taxonomically identified as a *Tityus* sp. Only documents confirming the species identity of scorpions responsible for envenomations were included in the full review process. Reports of envenomation cases where the scorpion species was not identified, or was presumably *Tityus* sp., were either excluded or used only for the discussion. Inclusion and exclusion of documents was assessed independently by two of the authors, and discrepancies were resolved by consensus. Database searches retrieved 777 candidate documents; elimination of duplicates yielded 583 unique records in English, Spanish, or Portuguese. Assessment of titles, abstracts and texts, and the evaluation of documents against inclusion criteria identified 14 reports for full data extraction [8, 11, 12, 14, 15, 30, 43, 44, 45, 46, 50, 51, 52, 53]. Figure 4 presents the flow diagram of the above review process.

Clinical manifestations from the abovementioned reports are summarized in Table 2. Envenomation syndromes were reported from the Amazon regions of Brazil, Ecuador, Venezuela, and French Guiana, for a total of 368 envenomation cases during the period 1950 to 2020. Seven papers analyzed clinical manifestations after envenomation by *Tityus obscurus* [11, 12, 46, 50, 51, 52, 53] (from Pará state, Brazil, and Cayenne, French Guiana), three by *T. silvestris* (Pará and Amazonas states, Brazil) [8, 44, 45], two by *T. apiacas* (Amazonas state, Brazil) [8, 43], and one each by *T. metuendus* (Amazonas state, Brazil) [8], *T. raquelae* (Amazonas state, Brazil) [8], *T. strandi* (Pará state, Brazil) [30], *T. breweri* (Bolívar state, Venezuela) [15], and *T. cisandinus* (from Morona Santiago, Ecuador) [14]. Scorpions involved in human injuries from Amazonian Ecuador reported by Roman *et al*. [14] which were not identified at that time, but were recently identified by one of the authors as *Tityus cisandinus* [54].

**Table 2.** Scorpion envenomation cases recorded in the Amazon region with positively identified associated species in the genus *Tityus* in the period from 1950 to 2020.

Parameters/references	Gomes *et al.* [8]	Gomes *et al.* [8]	Gomes *et al.* [8]	Gomes *et al.* [8]	Pardal *et al*. [51]	Pardal *et al.* [46]	Pardal *et al.* [46]	Pardal *et al.* [52]	Torrez *et al.* [53]	Kallel *et al.* [12]	Floch *et al.* [50]	Hommel *et al*. [11]	Monteiro *et al*. [45]	Coelho *et. al*. [44]	Silva *et al.* [43]	Silva de Oliveira *et al*. [30]	Román *et al.* [14]	Borges *et al.* [15]
**Scorpion species**	*Tityus metuendus*	*Tityus silvestris*	*Tityus raquelae*	*Tityus apiacas*	*Tityus obscurus*	*Tityus obscurus*	*Tityus obscurus*	*Tityus obscurus*	*Tityus obscurus*	*Tityus obscurus*	*Tityus obscurus*	*Tityus obscurus*	*Tityus silvestris*	*Tityus silvestris*	*Tityus apiacas*	*Tityus strandi*	*Tityus cisandinus*	*Tityus breweri*
**Number of cases**	103	22	12	7	72	14	34	1	58	1	1	1	1	13	4	3	20	1
**Country**	Brazil	Brazil	Brazil	Brazil	Brazil	Brazil	Brazil	Brazil	Brazil	French Guiana	French Guiana	French Guiana	Brazil	Brazil	Brazil	Brazil	Ecuador	Venezuela
**State/Province**	Amazonas	Amazonas	Amazonas	Amazonas	Western Pará	Western Pará	Eastern Pará	Eastern Pará	Western Pará	Cayenne	Cayenne	Cayenne	Amazonas	Eastern Pará	Amazonas	Western Pará	Morona, Santiago	Eastern Bolívar State
**Manifestations at the sting site**																		
Dry sting	-	-	-	-	-	-	+	-	-	-	-	-	-	-	-	-	-	-
Paresthesia	+	+	+	-	+	+	+	-	-	-	-	-	+	+	-	+	+	-
Pain	+	+	+	+	+	+	+	+	-	-	+	+	+	+	+	+	+	+
Irradiating pain	-	-	-	-	+	+	+	-	-	-	-	-	-	-	-	-	-	+
Edema	+	+	+	-	+	+	+	+	-	-	-	-	+	+	+	+	+	-
Erythema	+	+	+	+	-	+	+	+	-	-	-	-	-	+	+	+	-	+
Burning sensation	-	-	-	-	+	+	-	-	-	-	-	-	-	-	-	+	-	-
Ecchymosis	-	-	-	-	+	-	-	-	-	-	-	-	-	-	-	-	-	-
Piloerection	+	-	-	+	+	+	-	-	-	-	-	-	-	-	-	-	-	+
Sweating	+	-	-	+	-	+	-	-	-	-	-	-	-	-	-	-	-	-
**General manifestations**																		
Vertigo	-	-	-	-	-	-	-	-	-	-	-	-	-	-	-	+	-	-
Prostration	-	-	-		+	+	-	+	-	-	-	-	-	+	-	-	-	-
Sweating	+	-	-	+	+	+	+	+	+	-	-	-	-	-	+	-	+	+
Tremors	+	+	-	-	+	+	-	-	-	-	-	-	-	-	+	+	-	+
Asthenias	-	-	-	-	+	-	+	-	-	-	-	-	-	-	-	+	-	-
General unrest	-	-	-	-	-	-	-	-	-	-	-	-	-	+	-	-	-	-
Goosebumps	-	-	-	-	-	+	-	-	-	-	-	-	-	-	-	-	-	-
Paleness	-	-	-	-	-	-	-	-	-	-	-	-	-	-	+	-	-	-
Priapism	-	-	-	-	-	-	-	-	+	-	-	-	-	-	-	-	-	-
Myalgia	-	-	-	-	-	-	-	-	-	-	-	-	-	-	-	-	+	-
Peripheral cyanosis	-	-	-	-	-	-	-	-	-	-	-	+	-	-	-	-	+	-
Lethargy	+	-	-	-	-	-	-	-	-	-	-	-	-	-	-	-	-	-
Fever	-	-	-	-	+	-	-	-	-	-	-	+	-	-	-	-	+	+
Rhinorrhea	-	-	-	-	-	-	-	-	-	-	-	-	-	-	-	-	-	+
**Gastrointestinal manifestations**																		
Sialorrhea	+	-	-	-	+	+	-	+	+	+	+	+	-	-	-	-	+	+
Nausea	+	+	-	+	+	+	+	-	-	-	-	-	-	+	-	+	+	-
Vomiting	+	-	-	+	+	+	-	+	-	-	-	+	-	+	+	+	+	-
Diarrhea	+	+	-	-	-	-	-	-	-	-	-	-	-	-	+	-	-	-
Abdominal pain	+	+	-	-	-	+	-	-	-	+	-	+	-	-	-	-	-	-
Hiccup	-	-	-	-	-	-	-	-	-	-	+	-	-	-	-	-	-	-
Odynophagia	-	-	-	-	-	-	-	-	-	-	-	-	-	-	-	-	-	+
**Cardiorespiratory manifestations**																		
Coughing	-	-	-	-	-	-	-	-	-	-	-	-	-	-	-	-	-	+
Dyspnea	+	-	-	-	-	-	-	-	-	-	+	-	+	-	-	-	+	-
Tachycardia	+	+	-	-	+	-	-	+	+	+	-	+	+	-	-	+	+	+
Bradycardia	-	-	-	-	-	-	-	-	-	-	-	-	-	-	-	-	+	-
Hypertension	-	-	-	-	+	+	-	-	+	-	+	-	+	-	-	-	-	-
Hypotension	+	-	-	-	-	-	-	-	-	-	-	-	-	-	-	-	-	-
Tachypnea	+	+	-	-	-	-	+	+	-	+	-	+	-	-	-	-	+	+
Arrhythmia	-	-	-	-	-	-	-	-	-	-	-	-	-	-	-	+	-	-
Respiratory insufficiency	+	-	-	-	-	-	-	-	-	-	-	+	-	-	-	-	-	-
**Ophthalmological manifestations**																		
Blurry vision	+	-	-	-	-	+	+	-	-	-	-	-	-	-	-	-	-	-
Conjunctival hyperemia	-	-	-	-	-	+	-	-	-	-	-	-	-	-	-	-	-	-
Photophobia	-	-	-	-	-	+	-	-	-	-	-	-	-	-	-	-	-	-
Miosis	-	-	-	-	-	-	-	-	-	-	-	-	-	-	+	-	-	+
Visual hallucinations	-	-	-	-	+	-	-	+	-	-	-	-	-	-	-	-	-	-
Tearing	-	-	-	-	-	-	-	-	-	-	-	-	-	-	-	-	-	+
**Neurological manifestations**																		
Headache	+	+	+	-	-	-	+	-	-	-	-	-	+	-	-	-	+	-
Drowsiness	-	-	-	-	+	+	+	+	+	+	-	-	+	+	-	-	+	-
Dizziness	-	-	-	-	-	+	-	-	-	-	-	-	-	-	-	-	-	-
Mental confusion	-	-	-	-	+	+	-	-	-	-	+	-	-	-	-	-	-	-
Agitation	+	+	-	-	+	+	-	-	+	+	+	+	+	-	+	-	-	-
Whole body paresthesia	-	-	-	-	+	+	-	-	-	-	-	-	-	-	+	-	-	-
Myoclonus	_+_	+	-	+	+	+	-	-	+	-	+	-	+	-	-	-	-	-
“Electric shock” sensation	-	-	-	-	+	+	-	-	+	-	-	-	-	-	-	+	-	-
Dysmetria	-	-	-	-	+	+	-	-	+	-	-	-	-	-	-	-	-	-
Dysarthria	-	-	-	-	+	+	-	-	+	+	-	-	-	-	-	-	+	-
Ataxic march	-	-	-	-	+	+	-	-	+	-	-	-	-	-	-	+	-	-
Hyperreflexia	-	-	-	-	+	-	-	-	+	-	-	-	-	-	-	-	-	-
Spasticity	-	-	-	-	+	-	-	-	-	-	-	-	-	-	-	-	-	-
Hyperalert	-	-	-	-	+	-	-	-	-	-	-	-	-	-	-	-	-	-
Muscle fasciculations	-	-	-	-	-	+	-	-	+	-	-	-	-	-	-	-	+	+
Motor incoordination	-	-	-	-	-	+	+	-	-	-	-	-	-	-	-	-	-	-
Convulsions	+	-	-	-	-	-	-	-	-	-	-	-	-	-	-	-	+	-
Coma	-	-	-	-	-	-	-	-	-	-	+	-	-	-	-	-	-	-
Romberg signal	-	-	-	-	+	-	-	-	+	-	-	-	-	-	-	-	-	-
Babinski signal	-	-	-	-	+	-	-	-	-	-	-	-	-	-	-	-	-	-
**Severity**																		
Class I	85	19	12	0	17	5	26	0	15	0	0	0	0	10	0	1	1	0
Class II	14	2	0	7	55	9	6	0	28	0	1	0	0	3	4	2	18	1
Class III	4	1	0	0	-	-	-	1	15	1	0	1	1	0	0	0	1	0
Class IV (Deaths)	0	0	0	0	0	0	0	0	0	0	0	1	0	0	0	0	1	0

Local clinical manifestations (at the sting site) occurred in 88.8% of the cases, with pain and edema being the most frequent. 61.1% of the cases presented with systemic clinical manifestations, including cardiorespiratory (61.1%), general, gastrointestinal, and neurological (55.5%), and ophthalmological alterations (16.6%). Among the general manifestations, most frequent signs and symptoms were sweating and tremors. Main gastrointestinal manifestations were vomiting, nausea, and sialorrhea. Cardiorespiratory alterations included tachycardia and tachypnea. Ophthalmological manifestations included blurred vision and visual hallucination. Neurological alterations included agitation, somnolence, and myoclonus.

Regarding severity (based on the international consensus scale for classification of scorpion sting severity) [55], 52% (n = 10 cases) corresponded to Class I, 40.7% (n = 13) to Class II, 6.7% (n = 8) to Class III, and 0.5% (n = 2) to Class IV, which refer to fatal outcomes caused by *Tityus obscurus* in French Guiana and *T. cisandinus* in Ecuador.

Stings by *T. raquelae* did not present with systemic manifestations and therefore envenomation by this species is classified as Class I. On the contrary, stings by the remaining analyzed *Tityus* spp., including taxa from outside the Brazilian Amazonia, presented with systemic manifestations and severities in Classes I to IV, predominantly including neurological complications. In the case of *T. obscurus*, most frequent alterations were somnolence, agitation, myoclonus, dysarthria, “electric shock” sensation, dysmetria, and gait ataxia. In at least one *T. obscurus* case, generalized myoclonus persisted for more than 3 days, and was refractory to the use of diazepam [53]. In a sting case by *T. apiacas*, myoclonus was the main manifestation, whereas the primary symptoms after a sting by *T. silvestris* were headache, agitation, and myoclonus. *T. strandi* stings mainly produced an “electric shock” sensation and gait ataxia. Summarizing shared and species-specific manifestations, myoclonus have been described for *T. obscurus*, *T. apiacas*, and *T. silvestris*; dysmetria and Romberg signal has only been described in the case of *T. obscurus*, whereas dysarthria has been reported after envenomation by *T. obscurus* and *T. cisandinus*; gait ataxia for *T. obscurus* and *T. silvestris*. The “electric shock” sensation has been reported for *T. obscurus* and *T. strandi*.

Autonomic (sympathetic and parasympathetic) manifestations, instead of neurological, are predominant after envenomation by southern South America *Tityus*, such as *T. serrulatus* and *T. trivittatus* [56, 57], and also by northern Venezuelan scorpions *T. discrepans* and *T. zulianus* [58], Colombian *Tityus pachyurus* and *T. asthenes* [59, 60], and Trinidadian *T. trinitatis* [61]. While clinical consequences of peripheral neurotransmitter exacerbation have also been observed after envenomation by Amazonian scorpions, such as *T. obscurus* (e.g. pancreatitis [12]), neurological complications as seen in these cases are uncommon in South America outside of Amazonia. These alterations are more typical of the envenomation by North American *Centruroides* spp. [62, 63] and South African *Parabuthus* spp., particularly *P. transvaalicus* [64].

Our analysis underscores the fact that neurological manifestations are present in cases throughout the Amazon region and not just in Brazil, which may underlie common physio-pathological mechanisms among toxins produced by Amazonian *Tityus* spp. For instance, Folch *et al*. [50] were first to record in 1950 the “electric shock” sensation in a patient stung by *T. cambridgei* (later synomyzed with *T. obscurus*) in French Guiana. Although not included in our analysis (the scorpion was not identified), reports on another scorpion envenomation from French Guiana indicate that it presented with right hemiparesthesia and neurogenic bladder, the latter possibly a result of the onset of a conus medullaris syndrome [65]. Neurological manifestations, including tonic-clonic convulsions, have also been reported after scorpion stings in areas inhabited by *T. perijanensis* [38], a species inhabiting the Colombia/Venezuela border, which has been shown to produce sodium channel-active toxins structurally related to toxins from *T. obscurus* and *T. metuendus* [33]. Muscle fasciculations were recorded after *T. breweri* envenomation in Amazonian Venezuela, and by a *Tityus* spp. in Huila, southern Colombia (Imeri area of endemism), which is a manifestation not usually observed after stings by *Tityus* spp. in those countries, and is reminiscent of similar effects in the case of *T. obscurus* [15, 16] (Table 2).

## Physiopathology of Amazonian scorpion envenomation

The physiopathological mechanism of envenomation by Amazonian *Tityus* spp., particularly *T. obscurus* that explains its clinical outcome has not yet been fully elucidated. However, growing toxicological and physiological evidence suggests out that it differs significantly from the autonomic exacerbation mechanism reported for other buthid scorpions.

Three physiological paths of phenomena have been invoked to explain neurological complications after envenomation by scorpion species mainly producing autonomic manifestations. First, cerebral damage can be elicited by excessive blood pressure because of the scorpion toxin-induced peripheral massive release of catecholamines [66]. Also, brain ischemia could result from a defect in oxygen transport secondary to the pulmonary edema and cardiogenic shock observed in severely envenomed patients [67]. A third hypothesis is the direct action of scorpion toxins on the central nervous system (CNS). In this sense, Clot-Faybesse *et al*. [68] have suggested that neurological sequelae in envenomed infants are a consequence of their blood-brain barrier being significantly more permeable to scorpion toxins than adults. The fact that no significant passage of scorpion toxin of the blood-brain barrier occurs in adult mice and rats has been taken to indicate that CNS toxicity in adults is likely the result of the venom’s peripheral action, at least in the case of toxic Old World species (e.g. genera *Androctonus* and *Leiurus*) [69, 68]. As for Amazonian *Tityus* spp. producing neurological alterations in both infants and adults, where the appearance of adrenergic/cholinergic stimulation is less frequent or even absent, an alternative mechanism needs to be proposed. Torrez *et al*. [53] suggested that the neurological symptomatology presented after *T. obscurus* envenomation is the result of an acute cerebellar dysfunction together with abnormal neuromuscular manifestations. Silva de Oliveira *et al*. [30] proposed the possibility that these venoms, particularly *T. obscurus*, contain toxins with a higher affinity for ion channels expressed on sensory neuronal membranes (pain and sensation of “electric shock” in *T. obscurus* and *T. strandi*), motor neurons (hypertonia, fasciculation, myoclonus and spasm) and in CNS neurons (ataxia), with less affinity for ion channels expressed by peripheral neurons. In support of the latter possibility, it is known that scorpion toxins active on sodium channels (the most lethal components of buthid scorpion venoms) exhibit exquisite specificity towards channel´s subtypes [70], and that there is a differential expression pattern of such subtypes across nervous system tissues [71]. For instance, toxin LqhII from *Leiurus haebreus* is more potent towards sodium channel subtypes expressed in peripheral nervous system compared with LqhIII, from the same venom, which is significantly more active towards subtypes expressed in the brain [72].

Regarding the proposed action at the cerebellar level of *T. obscurus* and possibly other Amazonian *Tityus* toxins, no direct evidence is available at present. Significantly, recent work has suggested the importance of cerebellar neuronal dysfunction resulting from mutations in specific ion-channels that regulate membrane excitability in the pathogenesis of cerebellar ataxia in humans [73]. While various channelopathies in both sodium and potassium channels have been associated to cerebellar ataxia, some forms of gait ataxia have been recently linked to loss-of-function mutation on the BK (large conductance, calcium-activated potassium) channel which affects its selectivity filter reducing channel conductance and ion selectivity [74]. In this sense, CNS-specific potassium and/or sodium channel toxins may be produced by Amazonian *Tityus*, particularly in the *T. obscurus* group (e.g *T. obscurus* and *T. cisandinus*), that could account for the cerebellar alterations, including gait ataxia, which could act synergistically with other toxin types on specific channel subtypes expressed in motor and sensory neurons. If such *T. obscurus* CNS-specific toxins exist, a mechanism has to be postulated to explain their crossing of the blood-brain barrier in adult individuals.

Pulmonary edema, generally the cause of death induced by buthid scorpion venoms (including *T. serrulatus*), can have a cardiogenic origin as a result of the venom-induced massive release of catecholamines and depressed left ventricular systolic function, and also a noncardiogenic component, related to increased vascular permeability due to the activation of inflammatory mediators [75]. In fact, a cytokine storm initiates inflammatory-induced multi-organ dysfunction, often leading to an acute respiratory distress syndrome [76]. In rats, *T. obscurus* venom (10 mg/kg, i.p.) causes hemorrhagic patches in lung parenchyma but does not lead to lung edema [47]. The absence of edema in the case of *T. obscurus* implies the existence of a different mechanism of venom-elicited lung damage. Significantly, in the case of South African *P. transvaalicus* envenomation, where similar neuromuscular disturbances to *T. obscurus* have been reported, predominant cholinergic (instead of adrenergic) stimulation is protective of pulmonary edema [64]. It remains to be established in the case of *T. obscurus*, which neurotransmitters are associated with scorpion venom activity, generally resulting in presynaptic depolarization [77], and also which inflammatory mediators are produced as a result of the envenomation for a better understanding of the aetiology of lung disturbances in these cases.

Pulmonary disturbances vary across Amazonia, as cases by *T. metuendus* and the *T. obscurus* population inhabiting the area of Cayenne (French Guiana) present with dyspnea and respiratory insufficiency [8, 11], whereas envenomation by *T. obscurus* and *T. silvestris* from Pará, Brazil, do not [46, 44]. This suggests that different mediators associated to lung damage may be involved because of species- and/or population-specific toxinological differences across the Basin. Particularly, manifestations after *T. metuendus* envenomation, the species of greatest epidemiological importance in the Manaus region, resemble that of *T. serrulatus* and *T. bahiensis* in the Brazilian southeast [8], although 6% of the cases presents with myoclonia, a feature lacking by the latter species´ envenomation. Pediatric cases of scorpion envenomation in areas of French Guiana (where *Tityus* spp. are prevalent, including *T. obscurus*) present with a combination of cholinergic (including bradycardia, hypersecretion, and bronchoconstriction) and adrenergic (e.g. tachycardia, seizures) manifestations, in addition to neurological alterations [9]. Leukocytosis and hyperglycemia, known markers of poor prognosis in scorpionism [78, 79], are also present in these cases. These clinical alterations differ from the mainly central manifestations observed in cases from western Pará, Brazil, by *T. obscurus* [46]. The toxinological basis for such differences in clinical manifestations are not yet evident but comparative transcriptomic and phylogenetic studies, particularly across *T. obscurus* distribution, should throw light on whether population-specific toxin repertoires are produced despite the strong phylogenetic association predicted for Amazonian species (Figure 4). In this sense, our phylogeny demonstrates a 12.02% CO1 divergence (uncorrected p-distance) between eastern and western Brazilian *T. obscurus* populations, which may underlie differences in ion channel-specific toxin expression patterns as recorded in the case of populations of the Chinese scorpion *Lychas mucronatus* [80]. Molecular clock estimates suggest that the two populations shared a common ancestor in the Miocene, an evolutionary depth usually attributed to different species in scorpions [81, 82]. Thus, *T. obscurus* likely represents at least two morphologically similar but toxinologically disparate species.

Regarding effects at the muscular level, Borja-Oliveira et al. [83] showed that *T. obscurus* venom exerts a positive inotropic effect on mouse diaphragm; i.e. it is able to potentiate contractile force in directly stimulated curarized muscles (when neuromuscular transmission is abolished by the acetylcholine competitive antagonist D-tubocurarine), indicating that this venom contains factors that can efficiently increase contractile force by acting directly on the sarcolemma. On the contrary, D-tubocurarine prevented the inotropic effect of *T. serrulatus* venom in the same preparation, indicating that its muscular effect is acetylcholine-dependent. This is additional evidence that venom composition and activity in *T. obscurus*, and possibly in other related Amazonian species, differ significantly from other congeneric species responsible for severe scorpionism in South America, mainly *T. serrulatus*.

Altogether, more research needs to be directed towards elucidation of Amazonian *Tityus* envenomation physiopathology, especially considering that species and population-specific clinical trends are evident across the Amazon River basin. Fortunately, efforts have already been made to understand the biochemical and physiological mechanisms of action in isolated *T. obscurus* sodium channel toxins (see following section).

## Molecular, biochemical, and electrophysiological studies on Amazonian scorpion venoms

Research to uncover the composition and physiological activity of Amazonian *Tityus* venoms has focused on *T. obscurus* and lately on *T. metuendus*. The two species accounted for the majority of envenomation cases in urban areas, and the studies mostly evaluated ion channel specificity or biomedical applications of native or synthetically derived venom components.

###  Venom toxic components from Tityus obscurus 

Batista *et al*. [84-87] were the first to characterize the toxins produced by *T. cambridgei* (later synonymed with *T. obscurus*) from specimens collected at Marajó Island, Pará state, Brazil, using a combination of high performance liquid chromatography and mass spectrometry, with more than 60 isolated components. Of these, 26 peptides were characterized by mass spectrometry and physiologically as toxins targeting sodium or potassium channels. Guerrero-Vargas *et al*. [48] later proceeded with the molecular cloning of *T. obscurus* toxins (primary structure of fifteen putative sodium channel toxins was characterized) and performed phylogenetic analyses to uncover the relationships of *T. obscurus* sodium channel-active toxins and those from available congeneric species. They postulated that a strong cladistic separation exist between toxins from the northern part of the Amazon basin and those produced by congeneric scorpions inhabiting southeast South America. This result is corroborated by our phylogenetic results (Figure 4).

Oliveira *et al*. [88] performed the first transcriptomic analysis of *T. obscurus* venom, combined with a proteomic analysis. Results confirmed primary structures of previously identified components and concluded that a high abundance of metalloproteinases is followed by sodium and potassium channel toxins, which together with proteases are the most abundant components in the venom. In addition to ion channel-active toxins, scorpion metalloproteinases are important venom components as they have been shown to hydrolyze neuropeptides *in vitro*, releasing mediators that could interact with ion channels and promote indirect neurotoxicity [89]. The work by Oliveira *et al*. was able to identify several *T. obscurus* putative venom components such as the following: anionic peptides, antimicrobial peptides, bradykinin-potentiating peptide, cysteine rich protein, serine proteinases, cathepsins, angiotensin-converting enzyme, endothelin-converting enzyme and chymotrypsin like protein, proteinases inhibitors, phospholipases and hyaluronidases. Importantly, their work reported that while major secreted venom component classes are highly similar among *T. obscurus* and *T. serrulatus*, their individual toxin sequences are considerably divergent, confirming previous findings by Guerrero-Vargas *et al*. [48].

Recently, Dias *et al*. [90] determined that approximately 5% of crude *T. obscurus* venom is composed of short linear, non-disulfide-bridged peptides (NDBP). As opposed to disulfide-bridged peptides, responsible for the venom neurotoxic effects, NDPBs display a cationic amphipathic a-helical structure which allows ample antibacterial, antifungal, antiviral, and cytolytic activities [91]. They characterized 27 major peptides among *T. obscurus* NDBPs, which were sequenced, and thirteen were synthetized and functionally characterized. Some of the novel peptides showed similarity to hypotensins, potassium channel toxins and the allergen 5 protein, but most do not match any known toxin. Some of these peptides showed a moderate increase in nociceptive sensibility and edematogenic activity after intraplantar administration in mice, and have been suggested to act synergistically to alter rearing or locomotion in prey/predators of *T. obscurus* by potentiating inflammatory processes [90]. Chemically synthesized NDPBs ToAP3 and ToAP4 (derived from cDNAs coding for putative *T. obscurus* antimicrobial peptides) inhibit inflammatory responses, decreasing the production of various inflammatory mediators and modulating dendritic cells’ activation and maturation, avoiding exacerbated inflammatory reactions. Besides, ToAP3 showed antibacterial activity against *Mycobacterium massiliense* [92]. A novel enzyme inhibitor (ToPI1) has been isolated from *T. obscurus* venom, which specifically targets trypsin and undergoes head-to-tail cyclization upon enzyme binding. It has been proposed that this peptide and its derivatives could be used as activity based-probes to image trypsin activity in live animals and tissues. Although ToPI1 is related structurally to potassium channel toxins, its reduced activity against several of these channels suggest fewer adverse effects from their possible therapeutic application [93].

Table 3 presents native venom components from *T. obscurus* which primary structure and function has been determined to date. Work by Tibery *et al*. [94] and Duque *et al*. [95] have unveiled the sodium channel subtype specificity of neurotoxins To1 and To4. Toxin To1, previously shown to change sodium permeation in rat cerebellum granular neurons, has been classified as a β-toxin. This is because it mainly affects the gating activation component of human sodium channel isoforms hNav1.3 and hNav1.6, which are the subtypes mostly expressed on cerebellar granular cells [96]. To4 is also a β-toxin mainly affecting hNav1.1, hNav1.2, and hNav1.4 isoforms. This work, taken together with the electrophysiological characterization of other *Tityus* β-toxins, underscores the exquisite sodium channel subtype specificity among toxins from species across the geographical distribution of this genus. Different sodium channel-active toxins have different abilities to promote neurotransmitter release [97]. Such differential specificity may have physiopathological implications as there is a correlation between clinical manifestations and the type of neurotransmitter being released as a result of the envenomation process [6]. This remains to be explored in the case of *T. obscurus* and related species, particularly considering that autonomic manifestations are few or even absent, at least in the case of *T. obscurus* populations inhabiting Amazonia in eastern Brazil.

**Table 3.** Physiologically and structurally characterized native components from *T. obscurus* venom.

Component (UniProtKB/PDB)	Venom class/Peptide mass	Ascribed function	Reference
To1 (P60214)	NaTx (7403.5 Da)	Decreases sodium current in rat cerebellum granular cells; β-toxin affecting activation threshold of human isoforms Nav1.3, Nav1.6, insect BgNav1, and arachnid VdNav1. It reduces peak Na+ current	[84]
To2 (P60212)	NaTx (7318.4 Da)	α-toxin activity in F-11 cell lines	[85]
To3 (P60213)	NaTx (7384.4 Da)	Affects Na+ permeability in pituitary GH3 cells, similarly to α-scorpion toxins	[98]
To4 (P60215)	NaTx (7253.5 Da)	β-toxin affecting activation threshold of human isoforms Nav1.1, Nav1.2, and Nav1.6	[94]
Tc1 (P83243)	KTx (2446.4 Da)	It blocks reversibly Shaker BK(+)-channels; solution structure available	[86, 99]
Tc30 (P60210)	KTx (3871.8 Da)	Potent inhibitor of K+-currents in human T lymphocytes	[87]
Tc32 (P60211)	KTx (3521.5 Da)	Potent inhibitor of K+-currents in human T lymphocytes	[87]
ToPI1 (6MRQ_I)	Trypsin inhibitor (3806.9 Da)	Specifically targets trypsin over chymotrypsin; undergoes cyclization upon enzyme binding	[93]

NaTx, sodium channel-active toxins; KTx, potassium channel-active toxins.

Regarding *T. obscurus* toxins targeting potassium channels, three components have been isolated an evaluated. Toxin Tc1, a 23 amino acid-long, highly charged (30% positively charged residues) peptide, targets not only the *Shaker* B K+ channel but other voltage-gated potassium channels present in the brain [86]. Toxins Tc30 and Tc32 have a high affinity for Kv1.3 channels expressed on human T lymphocytes, although they are poor blockers of *Shaker* B K+ channel [87]. More *T. obscurus* toxins acting on K+ channels remain to be characterized, as the transcriptomic and proteomic analyses of Oliveira *et al*. [88] recovered the sequences of 33 components putatively targeting potassium channels. A plausible role for potassium channel-specific Amazonian toxins in cerebellar pathology also needs to be explored. These toxins generally target the channel´s selectivity filter [100], a structural region where disease-related mutations have been linked to motor disorders.

###  Venom toxic components from Tityus metuendus 

*T. metuendus* venom (from specimens collected in the Manaus region) has been recently studied using mass fingerprinting analysis, with the identification of over 200 distinct molecular mass components. At least 60 sub-fractions were recovered using high performance liquid chromatography and five purified peptides were sequenced by Edman degradation. An electrophysiological assay of whole *T. metuendus* soluble venom demonstrated the presence of both α- and β-scorpion toxin types. The gating processes of sodium channel subtypes hNav1.1, hNav1.2, hNav1.6, and hNav1.7 exhibited both alpha (current inactivation) and beta (current activation) effects, whereas venom modification of isoform hNav1.4 only showed a beta effect [101]. Importantly, *T. metuendus* venom contained a significant number of homologs to *T. obscurus* toxins belonging to bradykinin-potentiating peptide, potassium and sodium channel toxin venom families. This adds further support to the notion that scorpion toxins with a similar structural/functional fingerprint probably exist throughout Amazonia, supported by the phylogenetic relationships of species sequenced thus far, including *T. metuendus* (Figure 4).

## Antivenom neutralization efficiency and antigenicity of Amazonian scorpion toxic components

A major concern in the treatment of scorpion envenomation in the Amazon region has been the reduced neutralization capacity of some clinical manifestations by available antivenoms, combined with the limited access to these immunobiologicals in remote, rural areas of the basin. Prompt application of equine-derived scorpion antivenom (in combination with appropriate supportive measures) is a proven therapeutic tool worldwide as specific immunoglobulins effectively clear circulating scorpion venom antigens, particularly in patients where severe manifestations are yet to develop [1, 102]. In the case of Brazilian Amazonia, available antivenoms are those produced against *T. serrulatus* and an anti-arachnidic polyvalent serum against *Phoneutria* and *Loxosceles* spiders and *Tityus* venoms [8, 51, 53]. No antivenom is currently used in French Guiana against scorpion envenomation [9]. In Amazonian Venezuela, the anti-*Tityus discrepans* antivenom has been used to treat envenomation by *T. breweri* [15]. In the Shuar communities of Morona Santiago, Amazonian Ecuador, where envenomation by *T. cisandinus* is frequent, the clinical approach has relied on supportive treatment as no scorpion antivenom is available in Ecuador [14]. The same goes for remote jungle areas of Guyana [13]. Torrez *et al.* [53] have pointed out that the anti-*T. serrulatus* antivenom did not significantly reduce the severity of the cerebellar-muscular manifestations elicited by *T. obscurus* envenomation in the area of Santarem, Pará state, Brazil, with the need to resorting to benzodiazepines for treatment. Gomes *et al*. [8] also explained that neurological manifestations in patients stung by *T. apiacas* did not improve upon administration of the anti-*T. serrulatus* antivenom. In some cases of envenomation by *T. strandi*, the intensity and body distribution of the “electric shock” sensation did not subside with serotherapy using this antivenom [30]. However, muscle spasms manifested in 6% of *T. metuendus* envenomation cases from the Manaus region, and usually ended about 6-8 h after serotherapy [8].

The fact that the some of the neurological manifestations in *T. obscurus*, *T. apiacas*, and possibly *T. strandi*, are refractory to treatment with the Brazilian scorpion antivenom is indicative of the existence of different toxin antigenic epitopes in these Amazonian species. This is due to significant amino acid sequence divergence between ion channel specific toxins from Amazonian and southeast *Tityus* spp. [88, 48]. Interestingly, the actual recognition of the low molecular mass fraction of *T. obscurus* venom is negligible in immunoblots upon reaction with anti-*T. serrulatus* antibodies [88]. Such lower reactivity towards *T. obscurus* venom antigens, in comparison with *T. serrulatus*, *T. bahiensis*, and *T. stigmurus*, has also been demonstrated when using sera from the three different manufacturers of scorpion antivenoms in Brazil in ELISA assays [103].

### The need for specific scorpion antivenoms for the Amazon region

Only three anti-*Tityus* antivenoms are produced in Latin America [anti-T. serrulatus (three producing institutions in Brazil), anti-T. discrepans (Venezuela), and anti-T. trivittatus (Argentina)] for treating envenomations by at least 30 species of proven medical importance in this genus [6]. Borges *et al*. [33] proposed partitioning the *Tityus* fauna into four venom antigenic areas that could guide the use of currently available antivenoms or suggest the preparation of new antibodies, particularly in the case of Amazonia. Immunochemical, molecular (cDNA cloning), and phylogenetic data point out the existence of a distinct toxinological area encompassing morphologically related *Tityus* spp. (e.g all have two ventromedian keels in metasomal segments II to IV) inhabiting Lower Central America (LCA), Colombia, and the Amazon region. The stronger immunochemical recognition of the low molecular mass fraction of venoms from these origins by the Venezuelan (anti-*T. discrepans*), compared to the Brazilian anti-*T. serrulatus*, indicates conservation of linear epitopes among *T. discrepans* and LCA-Colombian-Amazonian species. Competitive ELISA assays involving soluble proteins from the same venoms also indicate that congeneric species from this region share native conformational epitopes with *T. discrepans* to a greater extent than with southeast Brazilian species, suggesting similar toxin surface chemistries. Both linear and conformational epitopes are known to be involved in antibody recognition of scorpion sodium channel toxins [104]. This affinity is further supported by the phylogenetic association of *Tityus* toxins in this group, where β-toxins from Venezuela, LCA and Amazonia cluster separately from β-toxins from southeast South America.

Homologs of *T. obscurus* To2, To3, To4, To8, and To11 are found throughout the LCA-Colombian-Amazonian region [33]. However, *in vivo* experiments have indicated that the amount of Venezuelan antivenom required for effective neutralization in the region might be greater than that required to neutralize venom from *T. discrepans* and allied Venezuelan species. Venom neutralization of *T. perijanensis*, a species that belongs to the Amazon region based on toxinological criteria, requires three times as much anti-*T. discrepans* antivenom to neutralize the control venom. Immunochemical data indicate that the anti-*T. discrepans* antivenom is the best available treatment for scorpionism in Amazonia. That said, the aim in serotherapy is the use of highly specific antibodies for neutralization of circulating venom antigens, so we anticipate the need to prepare new neutralizing antibodies against *Tityus* spp. inhabiting this region. Additionally, it remains to be determined whether the Venezuelan antibodies are capable of neutralizing neurological manifestations characteristic of scorpionism in the region. These new antidotes should be of particular help in French Guiana, Colombia, Ecuador, Peru, and throughout the Amazon region of Brazil.

### Conclusions

Much remains to be done in Amazonia in regard to the design of effective therapeutic tools to treat scorpionism. A multicentric approach involving research and medical facilities in all affected areas should prove rewarding. In this sense, a joint effort between scorpion biologists and toxinological/medical teams to keep ascribing clinical cases and venom components to confirmed species is clearly needed. Health workers seeking specific information on the control, prevention, and treatment of *Tityus* envenomations, mainly by Brazilian species, are suggested to review recent publications on the subject [5]. Concerning the possibility of producing a Pan-Amazonian scorpion antivenom, the observation that recognition of *Tityus* low molecular mass components from Venezuelan and Central American is greatly increased when a mixture of several *Tityus* venoms is used as antigen could guide future immunization protocols for the preparation of polyvalent therapeutic antibodies effective in the region [33]. In parallel, transcriptomic/proteomic studies of other medically important species could help demonstrate the degree of shared components along the basin. As a consequence of such studies, preparation of chimeric proteins containing epitopes from main toxic and immunogenic *Tityus* venom components of the LCA/Colombian/Amazonian corridor, as similarly designed for crotoxin [105], would be instrumental as representative antigens for the preparation of neutralizing antibodies with a broad range of efficacy.

*Tityus* is the most species rich scorpion genus, so it is perhaps unsurprising that their venoms are also exceptionally diverse. Fortunately, some patterns are beginning to emerge, and as demonstrated by this review, venom diversity is better understood when also considering phylogenetic relationships. Our molecular phylogenetic analysis of medically significant *Tityus* from Amazonia supports this stance, as Amazonian species with venoms that present unique neurological complications form a monophyletic group. Thus, some aspects of *Tityus* venoms exhibit phylogenetic signal, an outcome that can aid the development of antivenom treatments. Additional sampling of poorly sampled regions in Amazonia would further benefit our growing understanding of *Tityus* spp. and their venoms, especially if studied in a cross-disciplinary context that includes phylogenetics. Such an approach would undoubtedly reveal new scorpion species. Some of these will likely be related to the medically significant Amazonian *Tityus* reviewed in this study, a group that has probably inhabited the region’s rainforests since the Miocene.

## Supplementary material

The following online material is available for this article:

Additional file 1. Uncorrected p-distances (lower diagonal) and K2P corrected distances (upper diagonal) among COI sequences from medically important *Tityus* species.
