# Supplementary material for: Amazonian scorpions and scorpionism: integrating toxinological, clinical, and phylogenetic data to combat a human health crisis in the world’s most diverse rainforest
Source: J Venom Anim Toxins Incl Trop Dis. 2021 Nov 29;27:e20210028. doi: 10.1590/1678-9199-JVATITD-2021-0028 (PMC8629433; doi:10.1590/1678-9199-JVATITD-2021-0028)
Supplement: Additional file 1. [file 1678-9199-jvatitd-27-e20210028-s1.pdf]

J Venom Anim Toxins incl Trop Dis,

https://doi.org/10.1590/1678-9199-JVATITD-2021-0028

Supplementary to "Amazonian scorpions and scorpionism: integrating toxinological, clinical, and phylogenetic data to combat a human health crisis in the world's most diverse rainforest"

Additional file 1. Uncorrected p-distances (lower diagonal) and K2P corrected distances (upper diagonal) among COI sequences from medically important *Tityus* species.

	Taxon		**1**	**2**	**3**	**4**	5	6	7	8	9	10	11	12	13	14
**1**	**T_obscurus_ToW9_MF466179**		**0.000**	**0.134**	**0.089**	**0.124**	0.201	0.250	0.241	0.160	0.179	0.229	0.149	0.153	0.271	0.237
**2**	**T_obscurus_ToE1_MF466180**		**0.120**	**0.000**	**0.126**	**0.071**	0.205	0.264	0.234	0.165	0.191	0.259	0.172	0.160	0.247	0.267
**3**	**T_metuendus_KY982269**		**0.082**	**0.114**	**0.000**	**0.095**	0.189	0.241	0.233	0.149	0.179	0.229	0.131	0.157	0.262	0.245
**4**	**T_cisandinus_MF466178**		**0.111**	**0.067**	**0.088**	**0.000**	0.186	0.226	0.229	0.153	0.184	0.221	0.164	0.164	0.255	0.237
5	T_zulianus_Venezuela_AY586789		0.176	0.179	0.167	0.164	0.000	0.250	0.218	0.183	0.139	0.225	0.164	0.178	0.266	0.250
6	T_trivittatus_Paraguay		0.211	0.220	0.205	0.194	0.211	0.000	0.172	0.221	0.210	0.088	0.198	0.225	0.232	0.230
7	T_serrulatus_RP_Brazil_AY586809		0.205	0.199	0.199	0.196	0.188	0.152	0.000	0.209	0.189	0.161	0.171	0.198	0.238	0.217
8	T_perijanensis_Venezuela_AY586787		0.144	0.147	0.135	0.138	0.161	0.191	0.182	0.000	0.183	0.238	0.150	0.192	0.233	0.224
9	T_discrepans_Venezuela_AY586796		0.158	0.167	0.158	0.161	0.126	0.182	0.167	0.161	0.000	0.179	0.152	0.190	0.225	0.249
10	T_trivittatus_Argentina		0.196	0.217	0.196	0.191	0.194	0.082	0.144	0.202	0.158	0.000	0.202	0.206	0.244	0.238
11	T_asthenes_Panama_MF466176		0.135	0.152	0.120	0.147	0.147	0.173	0.152	0.135	0.138	0.176	0.000	0.058	0.250	0.225
12	T_asthenes_Esmeraldas_Ecuador_MF466177		0.138	0.144	0.141	0.147	0.158	0.194	0.173	0.167	0.167	0.179	0.056	0.000	0.275	0.258
13	C_noxius_Mexico		0.226	0.208	0.220	0.214	0.223	0.196	0.202	0.199	0.194	0.205	0.211	0.229	0.000	0.115
14	C_infamatus_Mexico		0.202	0.223	0.208	0.202	0.211	0.196	0.188	0.194	0.211	0.202	0.194	0.217	0.106	0.000

Amazonian taxa are in bold.
